# High-altitude adaptation is accompanied by strong signatures of purifying selection in the mitochondrial genomes of three Andean waterfowl

**DOI:** 10.1371/journal.pone.0294842

**Published:** 2024-01-03

**Authors:** Allie M. Graham, Philip Lavretsky, Robert E. Wilson, Kevin G. McCracken

**Affiliations:** 1 Eccles Institute for Human Genetics, University of Utah, Salt Lake City, UT, United States of America; 2 Department of Biological Sciences, University of Texas at El Paso, El Paso, TX, United States of America; 3 School of Natural Resources and Nebraska State Museum, University of Nebraska–Lincoln, Lincoln, NE, United States of America; 4 Department of Biology, University of Miami, Coral Gables, FL, United States of America; 5 Department of Marine Biology and Ecology, Rosenstiel School of Marine, Atmospheric, and Earth Science, University of Miami, Miami, FL, United States of America; 6 Human Genetics and Genomics, University of Miami Miller School of Medicine, Miami, FL, United States of America; 7 University of Alaska Museum, University of Alaska Fairbanks, Fairbanks, AK, United States of America; Central University of Punjab, INDIA

## Abstract

Evidence from a variety of organisms points to convergent evolution on the mitochondria associated with a physiological response to oxygen deprivation or temperature stress, including mechanisms for high-altitude adaptation. Here, we examine whether demography and/or selection explains standing mitogenome nucleotide diversity in high-altitude adapted populations of three Andean waterfowl species: yellow-billed pintail (*Anas georgica*), speckled teal (*Anas flavirostris*), and cinnamon teal (*Spatula cyanoptera*). We compared a total of 60 mitogenomes from each of these three duck species (*n* = 20 per species) across low and high altitudes and tested whether part(s) or all of the mitogenome exhibited expected signatures of purifying selection within the high-altitude populations of these species. Historical effective population sizes (*N*_*e*_) were inferred to be similar between high- and low-altitude populations of each species, suggesting that selection rather than genetic drift best explains the reduced genetic variation found in mitochondrial genes of high-altitude populations compared to low-altitude populations of the same species. Specifically, we provide evidence that establishment of these three Andean waterfowl species in the high-altitude environment, coincided at least in part with a persistent pattern of negative purifying selection acting on oxidative phosphorylation (OXPHOS) function of the mitochondria. Our results further reveal that the extent of gene-specific purifying selection has been greatest in the speckled teal, the species with the longest history of high-altitude occupancy.

## Introduction

Oxidative phosphorylation (OXPHOS) refers to the synthesis of adenosine triphosphate (ATP) in the mitochondria, which is driven by the transfer of protons across the inner mitochondrial membrane via the electron transport chain (ETC). Mitochondrial-encoded OXPHOS and other nuclear-encoded genes involved in the ETC serve a critical function in the production of cellular energy fueled by the TCA cycle through substrate oxidation derived from carbohydrates, fatty acids, and proteins [1]. Consequently, mitochondria are constantly exposed to selective pressures as individuals adapt to the energetic requirements of different environments; and thus, are considered an important driver of speciation [2–4] as well as leading indicators of population divergence for studies involving historical phylogeography [5, 6]. To date, across lineages, many studies have identified adaptive mitochondrial variation associated with a physiological response to changes in oxygen (O_2_) availability, temperature, or oxidative stress, including differential mitochondrial gene expression and regulation [7–9], increased or decreased density of mitochondria in skeletal muscle [10, 11], examples of increased or decreased OXPHOS capacity and catalytic efficiency [12, 13], as well as positively-selected genetic variation [14–16]. In birds that have adapted to hypoxic, high-altitude conditions there also is ample evidence of selective pressures on mitogenomic protein coding regions [17–19]. Finally, adaptation to high-altitude environments where O_2_ availability is severely limited frequently has been shown to follow predictable paths of parallel or convergent evolution [20–23]. However, while this generally has been shown to be true for genes under strong directional selection like hemoglobin [24], this has not always been shown to be the case for studies of mitochondrial function in which much more variable responses generally have been revealed [13, 25–30].

While direct assessments of mitochondrial function are crucial for establishing causation, measuring of standing genetic variation offers another route to infer the process of natural selection. Standing genetic variation generally refers to the amount of genetic diversity present in a population before or after natural selection has occurred, and as such represents the diversity (or lack-thereof) that selection has to act upon. However, disentangling the roles that demography and selection play shaping standing genetic variation can be complicated, as the former can leave genomic signatures resembling the latter or vice versa, especially for a single non-recombining linkage group such as the mitochondrial DNA (mtDNA). This may be particularly important in the case of recent founder events and/or niche invasions, in which subsequent population expansion leads to a shift in the allele frequency spectrum resembling either positive directional selection, or yet another type of selection, purifying selection [31]. Purifying selection refers to the selective removal of deleterious alleles and is expected to play a pervasive role in maintaining the *status quo* of biochemical function through stabilizing selection. Like directional selection, purifying selection is also characterized by a reduction in standing genetic variation; thus, it is important to assess possible influences of both demography and selection when establishing whether particular genes or genetic regions show decreased standing variation due to any of these processes.

The Andes are the world’s highest mountains outside of Asia and possess some of the largest high plateaus in the world. In South America, the Altiplano is the largest such plateau extending from northwestern Argentina to southern Peru. The Altiplano contains many wetlands, including Lake Titicaca, that host a large number of waterfowl [32, 33]. Most prominent are the common dabbling duck (*Anas & Spatula*) species whose high-altitude populations are known to have independently colonized habitats in the Andes from closely-related ancestral low-altitude lineages [28]. To date, research on this taxonomic group has identified genetic convergence in both the hypoxia-inducible factor (HIF) pathway, as well as hemoglobin (Hb) genes [20, 22, 34, 35]. However, outside of these well-studied genes, genetic variation associated with other physiological mechanisms for high-altitude living remains relatively unexplored in this group. Here, we seek to determine whether convergent evolution resulting from either directional selection or purifying selection has played out in the mitogenomes of these same species of ducks due to its role in OXPHOS as described above.

We compared the standing genetic diversity of each low- and high-altitude population of three Andean duck species using ten mitogenomes per population. The species compared include the yellow-billed pintail *(Anas georgica*), speckled teal (*Anas flavirostris*), and cinnamon teal (*Spatula cyanoptera*; syn. *Anas cyanoptera*), which have been the focus of previous physiological studies spanning O_2_ transport [20, 22, 28, 36–41]. These paired low- and high-altitude population pairs provide natural replicates to study the genetic attributes linked to demography caused by historical founder events involving colonization of cold, high-altitude, hypoxic environments from ancestral low-altitude environments. These pairwise comparisons also represent contrasting depths of divergence times between the populations. Whereas respective low- and high-altitude populations of yellow-billed pintail and cinnamon teal diverged more recently, these mtDNA data reveal that speckled teal represent much deeper divergence by approximately two orders of magnitude [42–44]. Thus, these species allow us to infer potential differences caused by the effect of demography versus selective forces on standing genetic diversity among the population-pairs, including how historical colonization of the high-altitude environment impacted mitogenomic diversity either through directional selection or purifying selection. Ultimately, we find little evidence that variable demographic processes have shaped mitogenome diversity in these species. Instead, we present evidence consistent with the prevalence of stronger purifying selection in high-altitude populations compared to low-altitude populations of each species, with the strongest evidence for purifying selection occurring in the species that has occupied the high-altitude environment for the longest period of evolutionary time.

## Materials and methods

### Specimen collection and DNA extraction

**Fig 1:**
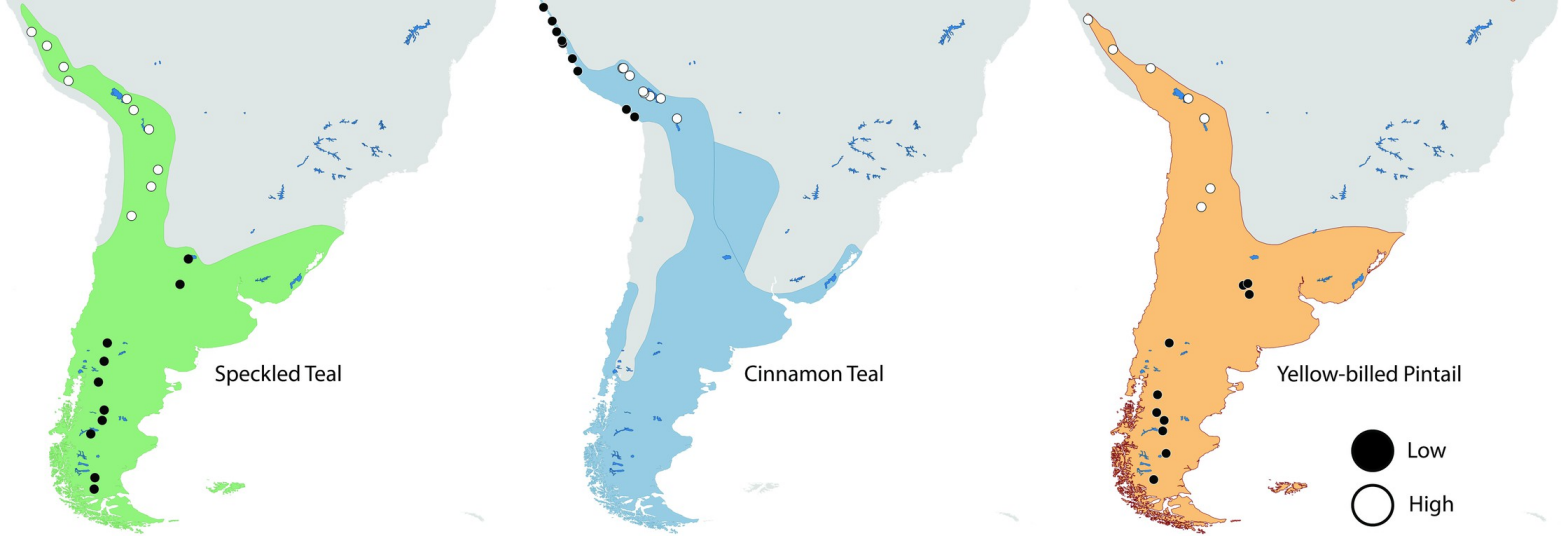
Species distribution of speckled teal (*Anas flavirostris*), yellow-billed pintail (*Anas georgica*), and cinnamon teal (*Spatula cyanoptera)* denoted by color ranges; individual sample collection locations are denoted by symbols. Species distribution map layers were obtained from [93].

A total of 60 individuals composed of 10 low- and 10 high-altitude samples across three species of ducks were used [22] (Fig 1). For the cinnamon teal, individuals from low-altitude populations are the *S*. *c*. *cyanoptera* subspecies (*n* = 10; elevation range 7–23 m) and from high-altitude are the *S*. *c*. *orinoma* subspecies (*n* = 10; elevation range 3533–3,871 m) [43]. For the speckled teal, individuals from low-altitude populations are the *A*. *f*. *flavirostris* subspecies (*n* = 10; elevation range 77–860 m) and from high-altitude are the *A*. *f*. *oxyptera* subspecies (*n* = 10; elevation range 3,211–4,405 m) [44]. For the yellow-billed pintail, individuals from both populations are taxonomically identified as the same subspecies *Anas georgica spinicauda*. A total of 20 yellow-billed pintails were collected from low- (*n* = 10; elevation range 292–914 m) and high-altitude (*n* = 10; elevation range 3,332–4,070 m) [42]. Genomic DNA was extracted from tissue using a DNeasy Tissue Kit (Qiagen, Valencia, California, USA) following manufacturers protocols.

### Target-enrichment sequencing

We utilized in-solution target capture to selectively enrich libraries for the complete mitochondrial genome prior to NGS sequencing [45]. All steps of the process were performed by MYcroarray (Ann Arbor, MI). In short, a custom MYbaits® biotinylated ssRNA target capture baitset was designed with 1,359 120mer probes at 2x tiling density from five previously published *Anas* mitochondrial genomes (*A*. *acuta*, NC_024631.1; *A*. *crecca*, KC771255.1; *A*. *formosa*, NC_015482.1, *A*. *platyrhynchos* NC_009684/EU009397.1; and *A*. *poecilrhyncha* NC_022418.1). DNA samples were then subjected to capture library synthesis and preparation. The prepared libraries were hybridized with the custom mitochondrial baits. Following hybridization, target regions were purified on magnetic beads followed by post-hybridization amplification to add indexing sequences. Ultimately, 250–300 bp insert sizes were targeted with sequencing of a single multiplexed library performed on an Illumina Hi-Seq platform using paired-end (100 bp) chemistry. Illumina reads were deposited in NCBI’s Sequence Read Archive (SRA; http://www.ncbi.nlm.nih.gov/sra; SRA data PRJNA508951).

### Mitochondrial genome assembly and annotation

All sequences were de-multiplexed by individual with adapters trimmed and quality filtered (Q < 30) post-sequencing using the FASTX-Toolkit v. 0.0.13 package [46]. Trimming was performed utilizing *fastq-clipper* (AGATCGGAAGAGC), and remaining sequences were then filtered by length and quality using *fastq-quality filter* (reads <20 bp, and Q < 30). The mitochondrial genome for each individual was assembled with MITObim v. 1.7 [47] using as a reference the full mitochondrial genome of the mallard (*Anas platyrynchnos*; EU009397.1), which is approximately equidistant from all study species to avoid a potential source of bias. For genome reconstruction, the “quick” and “trim” options were used, with an iteration limit set to 30. Mapping results were aligned with MAFFT [48] and annotated in Geneious v. 8.1.6 [49] based on 90% threshold similarity to the mallard reference. Protein coding, tRNA, and rRNA genes were extracted from these annotations and then subjected to individual analyses.

### Population genetic and selection analyses

All comparisons were made between low- and high-altitude populations within species. For each species, we calculated pairwise *F*_*ST*_ [50] for all variable sites for the full mitochondrial genomes as well as individual gene regions—protein coding, and non-protein coding (D-Loop, tRNAs, rRNA). This was done for each of the three species using Arlequin v. 3.5 [51] and MEGA v. 7 [52]. We also calculated Tajima’s *D* [53, 54], which compares the mean number of pairwise differences and number of segregating sites (e.g., Tajima’s θ vs. Watterson’s θ), scaled as such so that each are expected to be the same in a neutrally evolving population of constant size. Where this is not the case, *D* < 0 reveals a significant excess of high frequency polymorphisms and can be indicative of a recent selective sweep caused by directional selection or purifying selection (selection) and/or a founder event followed by population expansion (demography). *D* > 0 by contrast reveals a significant excess of both low- and high-frequency polymorphism and generally is indicative of either balancing selection (selection) or population contraction (demography).

Next, we utilized codon-based Z-tests to compare the relative abundance of synonymous and nonsynonymous substitutions in these mitochondrial sequences, where Z = (*d*_N_−*d*_S_) / √ (Var(*d*_S_) + Var(*d*_N_)). The null hypothesis (H_0_) is that the ratio is equal (*d*_N_ = *d*_S_), whereas *d*_N_ > *d*_S_ indicates directional (positive) selection and *d*_N_ < *d*_S_ indicates purifying (negative) selection. These analyses were performed estimating variance via a bootstrap method (500 replications) as implemented in MEGA v. 7 [54, 55].

To examine evidence for selection for specific codons, we used the SLAC (Single Likelihood Ancestor Counting) method, MEME (Mixed Effects Model of Evolution), and FUBAR (A Fast, Unconstrained Bayesian AppRoximation for Inferring Selection) through the program HyPhy v. 2.5 [56]. SLAC also assesses selection using the nonsynonymous/synonymous ratio (*d*N/*d*S), which is defined as ω, with values of ω < 1, ω = 1, and ω > 1 also signifying negative/purifying selection, neutral evolution, and positive/directional selection, respectively. However, these modules estimate ω at every codon in the alignments and report which codons show evidence of positive or negative selection, based on the expected and observed numbers of synonymous and nonsynonymous substitutions as inferred using maximum-likelihood [57]. Unlike SLAC, MEME allows ω to vary across codons as well as across branches of the phylogeny, allowing it to detect a small proportion of branches that are evolving under positive directional selection or under episodic selection [58]. To avoid a high false-positive rate, due to the reduced number of sequences, sites with *P* values < 0.1 for both models were considered significant [57]. Finally, FUBAR uses a Bayesian approach to infer nonsynoymous (*d*_N_) and synonymous (*d*_S_) substitution rates on a per-site basis for a given coding alignment and corresponding phylogeny, and assumes that the selection pressure for each site is constant along the entire phylogeny [59]. To avoid a high false-positive rate, due to the reduced number of sequences, sites with posterior probabilities > 0.9 for both models were considered significant.

### Demographic analyses ‐ population size fluctuation

Changes in effective population size (*N*_*e*_) across time were inferred using Bayesian Skyline plots as implemented in BEAST v. 1.8 [60]. We ran linear skyline models with a strict molecular clock, and optimum base-pair substitution models determined for each dataset based on Bayesian Information Criterion (BIC) scores as estimated in MEGA v. 10 [52]. Multiple chains were run for 100 million steps yielding effective sample sizes (ESS) of at least 200. The first 10% of runs were discarded as “burn-in”. All operators (transitional kernals or ‘moves’) in the Markov chain Monte Carlo were optimized automatically. Results of the analyses were illustrated using Tracer v. 1.5 [61].

### Divergence between low- and high-altitude populations

Finally, we calculated pairwise genetic divergence between each pair of low- and high-altitude populations. This was done for the full mtDNA and each protein coding gene listed above using the Tamura-Nei [62] model of substitution in MEGA v. 10 [52]. The purpose of this analysis was to compare relative divergence times between low- and high-altitude populations among the three species and therefore provide some perspective on the relative duration of occupancy of the high-altitude environment as recently examined in the context of biochemical changes in pathways of energy metabolism [28], in which species that had occupied the highlands for longer periods of evolutionary time had gradually shifted activities of key enzymes related to OXPHOS capacity relative to newcomers to the highlands.

## Results

### Assembly features and mitogenome organization

We attained an average sequencing depth of 232x across mitogenomes for all species. The final consensus genome sizes per species were 16,601bp (cinnamon teal), 16,602 bp (speckled teal), and 16,616 bp (yellow-billed pintail), respectively (S1 Table). Across these species, the light strand includes NADH6 and 8 tRNAs, whereas the heavy strand includes the remaining 12 protein coding genes, 14 tRNAs and the 12S and 16S rRNAs. The non-coding control region (D-loop) in these species is located between ND6/tRNA-Glu and the tRNA-Phe/12S ribosomal RNA genes similar to other waterfowl species [63]. Overall composition and arrangement of the mitochondrial genome is typical of birds and matches other Anseriformes species ranging from 16,608–16,594 bp in size [64–67].

**Fig 2:**
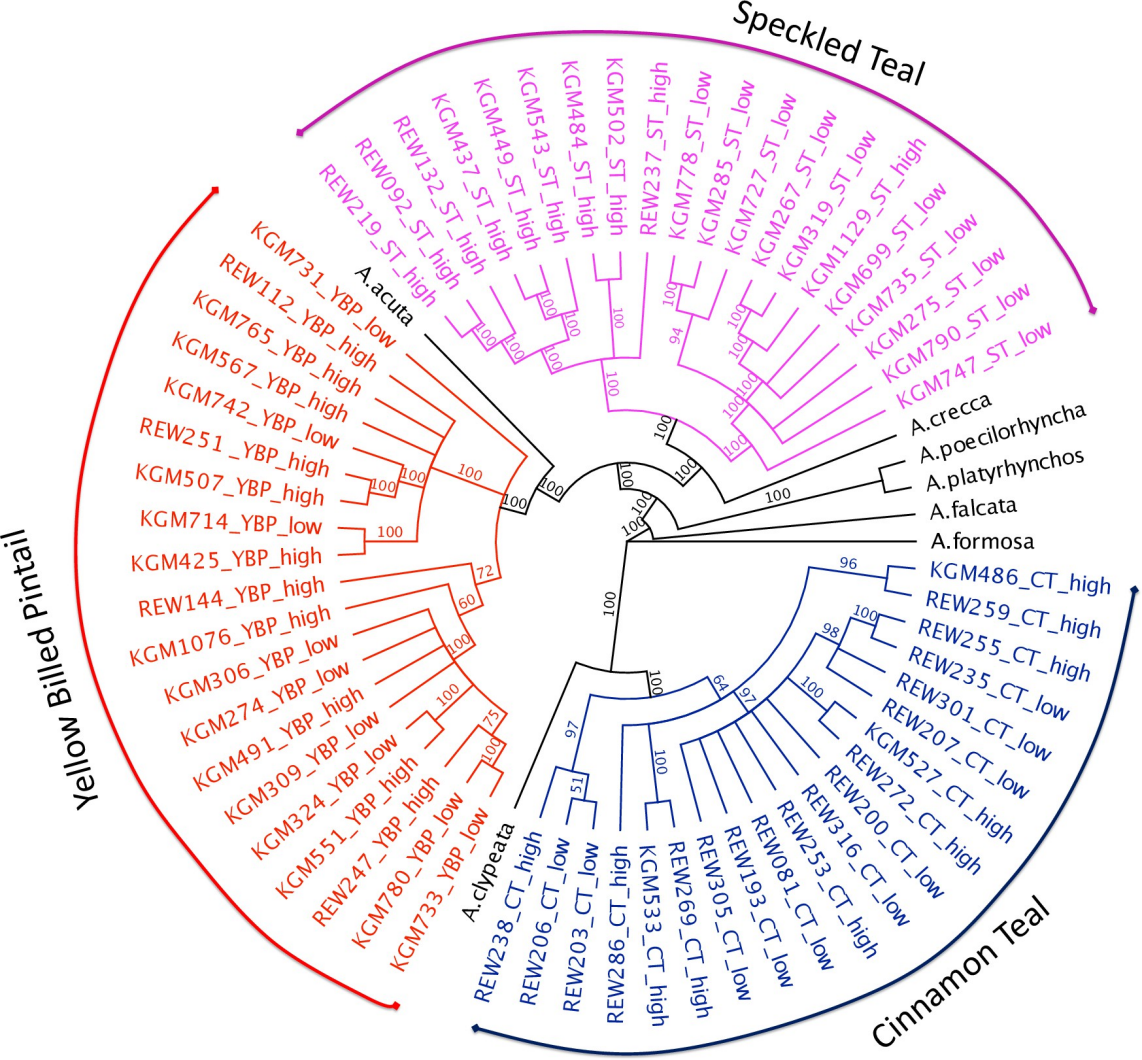
Maximum-likelihood tree (GARLI) using the full mitochondrial genomes of the speckled teal (pink), yellow-billed pintail (red) and cinnamon teal (blue), along with other *Anas* outgroups. Numbers at nodes represent percent consensus support; “high” = individuals from high-altitude populations, “low” = individuals from low-altitude populations.

A maximum-likelihood tree of all 60 individuals including the 6 outgroups (Fig 2) was constructed using GARLI [68, 69], in order to show general phylogenetic relationships between the species/populations. Outgroups for this analysis included *A*. *acuta* (NC_024631.1), *A*. *clypeata* (NC_028346.1), *A*. *crecca* (KC771255.1), *A*. *formosa* (NC_015482.1), *A*. *platyrhynchos* (EU009397.1), and *A*. *poecilrhyncha* (NC_022418.1).

### Demographic analyses ‐ population size fluctuation

**Fig 3:**
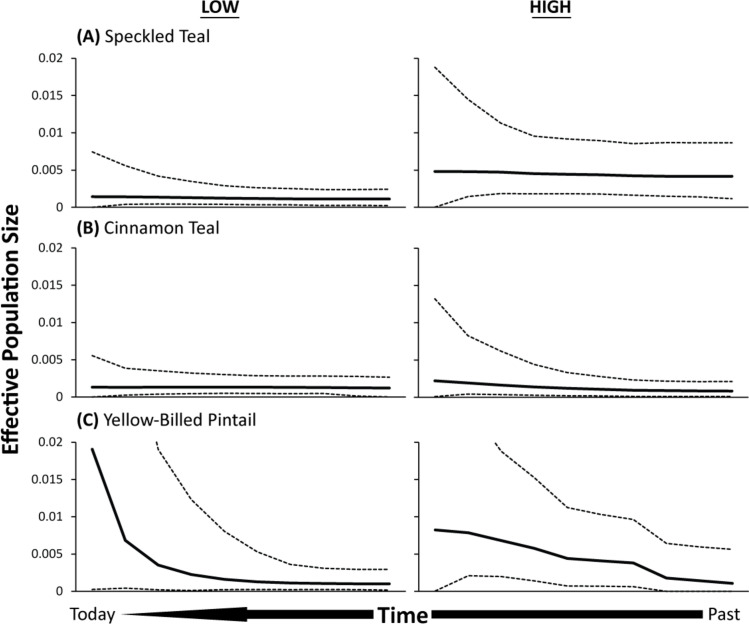
Bayesian skyline plots for low- and high-altitude populations of (A) speckled teal, (B) cinnamon teal, and (C) yellow-billed pintail.

Plotting the median and 95% confidence intervals revealed largely stable estimates of *N*_*e*_ across time for each analyzed low- and high-altitude population of speckled teal and cinnamon teal (Fig 3A and 3B). However, whereas speckled and cinnamon teal show almost constant effective population size through time, yellow-billed pintails appear to have more recently experienced exponentially increasing numbers, particularly in the case of the low-altitude yellow-billed pintail population (Fig 3C). The optimum base-pair substitution models for these analyses were determined to be either HKY85 or the similar TrN93 model.

### Population genetic analyses

**Fig 4:**
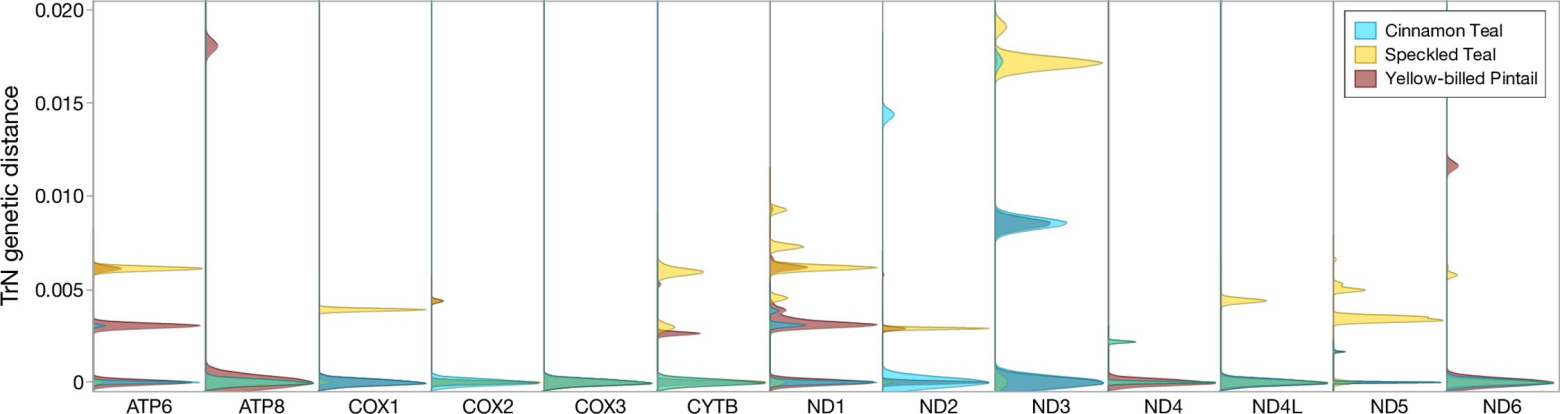
Kernal density plots showing pairwise distances between low- and high-altitude populations of each species calculated for each mitochondrial protein-coding gene using the Tamura-Nei (TrN) model of nucleotide substitution.

Comparisons within species between low- and high-altitude populations of cinnamon teal and yellow-billed pintail revealed extremely low mitochondrial differentiation, whereas comparisons within speckled teal revealed much higher differentiation for most mitochondrial gene regions, corresponding to approximately an order of magnitude deeper genetic divergence (Fig 4 and S2 Table), therefore reflecting a longer period of high-altitude occupancy by speckled teal.

For the cinnamon teal, the overall *F*_*ST*_ between low- and high-altitude was 0.02, with 64 segregating sites. For the yellow-billed pintail, *F*_*ST*_ between low- and high -altitude was 0.00, with 139 segregating sites. In contrast, *F*_*ST*_ between low- and high -altitude speckled teal was 0.71, with 206 segregating sites. In speckled teal, there were 119 polymorphisms that were fixed between low- and high-altitude populations, whereas no fixed differences occurred in the other two species. There were few instances of non-synonymous changes between high and low-altitude populations of cinnamon teal or yellow-billed pintail, and none with significant *F*_*ST*_ values. In cinnamon teal, there was a non-synomymous change in ND1 (Met160Val), ND4 (Ala163Thr), and none in yellow-billed pintail. Furthermore, SNPs that were significantly different in speckled teal (*P < 0*.*05*) were present in both noncoding (D-Loop, l-rRNA, tRNA-Ala, tRNA-Thr) and a large majority of protein coding genes; specifically, there were 5 instances of various non-synonymous changes resulting a high degree of fixation (*F*_*ST*_ > 0.75) in the speckled teal, specifically in ATP6 (Tyr13His), ND1 (Met325Thr), ND2 (Ile231Leu), ND3 (Met107Val), and ND5 (Ile128Val) (S3 Table). There were additional nonsynonymous changes with a moderate degree of fixation in speckled teal, including COX1 (Leu401Phe), ND2 (Met245Ile), ND3 (Pro76Leu, Val84Ile, Val107Met), and ND5 (Arg476Thr) (S3 Table).

### Tests for selection across the mitochondrial genome

Selection was also assayed at the level of specific codons using HyPhy [56], which uses ω (*d*_N_/*d*_S_ ratio). The intra-species SLAC analysis identified no credible instances of positive selection. The MEME analysis showed no evidence of episodic diversifying selection across any of the three species, whereas FUBAR showed extensive evidence of purifying selection in all three species across all genes (S4 Table). This same pattern arises when all three species data was combined, with widespread evidence of purifying selection, and no sites significantly under positive selection. Overall, the results from the three analyses (SLAC, MEME, FUBAR) identified many more examples of purifying selection, as compared to positive selection in all three species (S4 Table).

**Fig 5:**
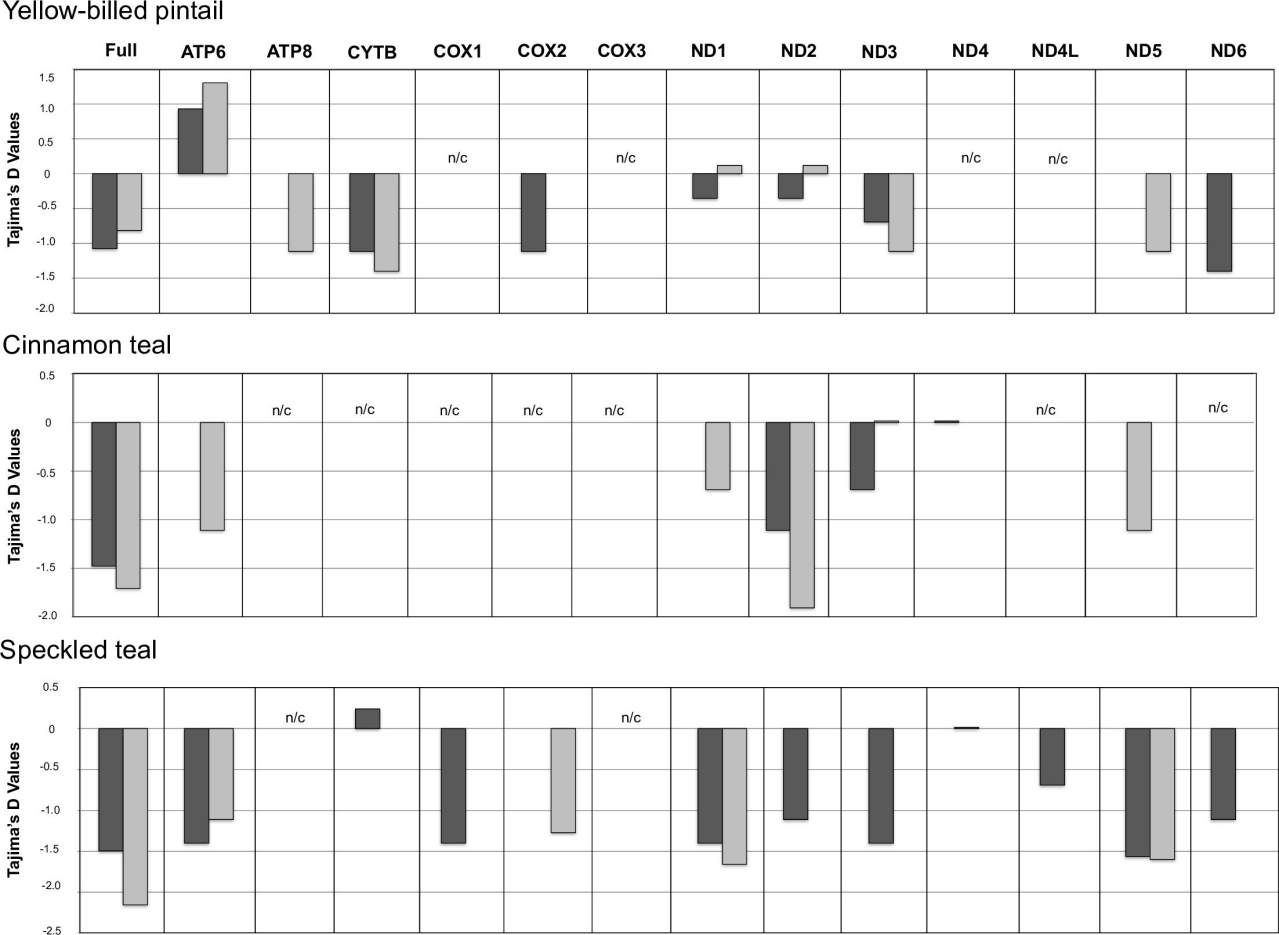
Tajima’s D for the individual protein-coding genes across each high- (dark grey) and low-altitude population (light grey) of the three species; nc = not calculated, due to a lack of nucleotide variation.

**Fig 6:**
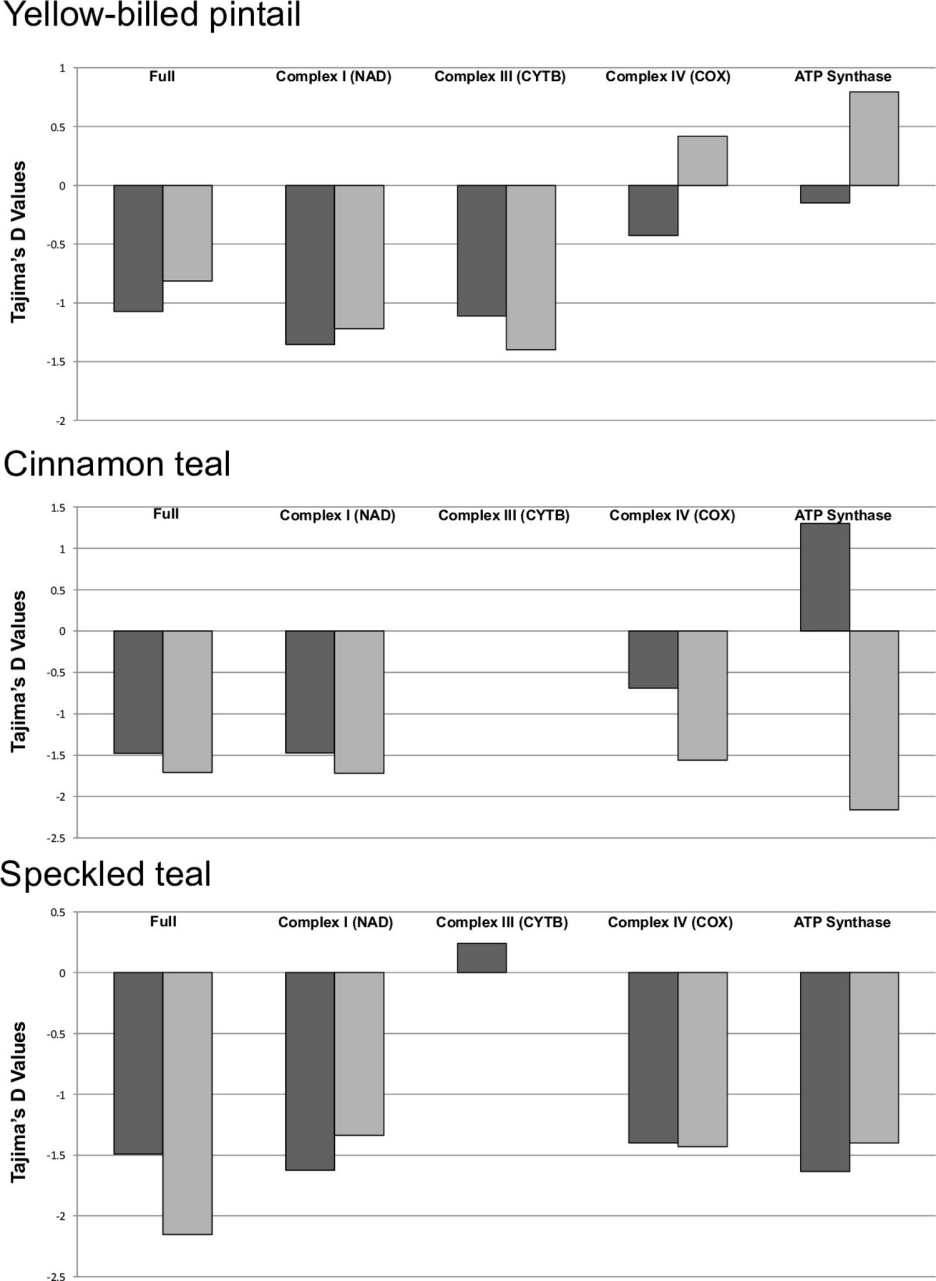
Tajima’s D for protein-coding genes across five of the mtDNA complexes in high- (dark grey) and low-altitude populations (light grey) of the three species.

Applying standard neutrality tests to the protein coding genes, both high- and low-altitude populations across species had significantly negative Tajima’s *D* estimates (Figs 5 and 6 and S5–S7 Tables). Specifically, cinnamon teal and yellow-billed pintail showed an excess of low-frequency SNPs, as well as paucity of high-frequency SNPs. By contrast, speckled teal showed an excess of low- and high-frequency SNPs segregating between the low- and high-altitude populations. Thus, the speckled teal showed a strikingly different site-frequency spectrum characteristic of a more deeply diverged pair of populations.

Finally, the codon-based Z-test was used to test hypotheses about the type of selection (neutral, positive, purifying) predominating between low- and high-altitude populations. For all three species, the Z-test for individual protein coding genes showed evidence for either neutrality or purifying selection (Table 1). None of the analyses in any of the three species yielded significant results for positive selection. However, the low-altitude speckled teal population showed significantly fewer genes under purifying selection, relative to the high-altitude speckled teal population (10; *X*^2^ = 7.539, *P < 0*.*01*). This pattern was not seen in either the cinnamon teal or the yellow-billed pintail (*P > 0*.*05*). However, comparing the incidence of sites under purifying selection across all low-altitude populations to all high-altitude populations showed evidence of more widespread purifying selection (*X*^2^ = 6.477, P *< 0*.*01*). In addition, COX2 and ND1 genes appear to be under purifying selection in the high-altitude populations across all three species, whereas the ND4-6 genes appear to be under purifying selection in both low- and high-altitude populations. Overall, neutrality tests with different sensitivities to demographic effects showed significant purifying selection in the protein coding OXPHOS genes of all high-altitude populations (Figs 5 and 6 and S5–S7 Tables).

**Table 1:** Codon-based Z-tests for positive (H_o_ = *d*_N_ > *d*_S_) and negative (H_o_ = *d*_N_ < *d*_S_) selection calculated across protein-coding gene regions; grey highlighted cells correspond to significant values (*P* < 0.05).

Species	Pop.	Selection Test	ATP6	ATP8	COX1	COX2	COX3	CYTB	ND1	ND2	ND3	ND4	ND4L	ND5	ND6
Yellow-billed pintail	High	Positive	0.304	1	1	1	1	1	1	1	1	1	1	1	1
	Low	Positive	0.493	1	1	1	1	1	1	1	1	1	1	1	1
	High	Purifying	1	0.064	0.028	0.011	0.044	0.27	0.156	0.009	0.35	0.003	0.086	0.012	0.001
	Low	Purifying	1	0.097	0.03	0.078	0.056	0.042	0.493	0.119	0.131	0.024	0.083	0.006	0.021
Cinnamon teal	High	Positive	1	1	1	1	1	1	1	0.145	1	1	1	1	1
	Low	Positive	1	1	1	1	1	1	1	1	1	1	1	1	1
	High	Purifying	0.146	1	0.067	1	1	1	0.048	1	0.395	0.007	1	0.049	0.042
	Low	Purifying	0.112	1	0.073	1	1	1	0.296	1	0.165	0.013	1	0.03	0.131
Speckled teal	High	Positive	1	1	1	1	1	1	1	1	0.73	1	1	1	1
	Low	Positive	1	1	1	1	1	1	1	1	1	1	0.147	1	1
	High	Purifying	0.03	0.146	0	0.039	0.001	0.027	0.001	0.007	1	0	0.098	0	0.006
	Low	Purifying	0.266	1	0.012	1	0.018	0.151	0.147	0.041	1	0.146	1	0.136	0.076

Because tRNA and rRNA genes do not produce an amino acid sequence, quantifying non-synonymous or synonymous mutations is not possible; thus, selection could only be assessed using Tajima’s D. Both tRNA and rRNA regions showed evidence of purifying selection and/or population expansion as compared to both the non-coding D-loop and protein-coding regions (S7 Table). Otherwise, each high-altitude population of each species showed patterns consistent with purifying selection.

## Discussion

In this study, we present a comparative analysis of mitogenome evolution in low- and high-altitude populations of three Andean waterfowl species that colonized the high Andes at different times in history. We performed various analyses aimed to disentangle the relative contributions of demographic and selective processes on the extant standing genetic diversity of each respective population’s mitogenomes. First, we found few or no observable changes in effective population size through time when comparing each respective high- versus low-altitude population within each of the three duck species (Fig 3). All populations within species were similar in this regard. Importantly, the lack of significant variation in effective population size between low- and high-altitude populations through time for each of the six lineages suggests that (1) all three species of these waterfowl did not experience strong reductions in genetic diversity following historical founder events in which the Andes were colonized from low-altitude ancestral populations, and (2) high-altitude populations continue to maintain their effective population size similar to the ancestral size. Given the lack of observable changes in effective population size between populations within species, we conclude that reductions in mitogenome variation of high-altitude populations have more likely been the result of stronger and more frequent instances of purifying selection than experienced by their low-altitude counterparts. In particular, we find evidence of the strongest purifying selection on the OXPHOS genes of the high-altitude populations, likely to be the result of selection for more efficient metabolism in high-altitude habitats [28, 70–72].

### Patterns of selection in the three Andean duck species

For the cinnamon teal and yellow-billed pintail, recent divergence has resulted in little or no difference between the high- and low-altitude populations in polymorphism across the entirety of the mitochondrial genome, which is consistent with their more recent demographic history in the highlands (Fig 4). However, the mitochondrial genomes of both species show evidence for purifying selection overall (Table 1 and Figs 5 and 6), with the strongest apparent negative selection being exerted on the coding regions for cinnamon teal, and noncoding regions (tRNAs, rRNAs) for the yellow-billed pintail. For the cinnamon teal, the high-altitude population is considered a separate subspecies, due to striking differences in plumage patterns and body size [73, 74], whereas low- and high-altitude populations of the yellow-billed pintail are classified as the same subspecies [42].

In contrast, the speckled teal showed the strongest evidence for purifying selection on standing mitochondrial genetic diversity. Despite a deeper divergence [44] between the high- and low-altitude populations, mitochondrial genome divergence was extreme (*F*_*ST*_ = 0.71) compared to nuclear DNA (*F*_*ST*_ = 0.06). In this species, our results suggest a pattern of non-neutral processes playing a role in divergence, which mirrors previous research on the evolution of Hb-O_2_ affinity across each of these species [20, 75, 76], as well as patterns of variation consistent with strong selection on the HIF pathway [22].

Altogether, our results also show a significant role for purifying selection in both low- and high-altitude populations of all three species, but especially in protein-coding regions of high-altitude populations (Figs 5 and 6 and Table 1, *X*^2^ = 6.477, *P < 0*.*01*). This implies that adaptation to hypoxic environments in these duck species has been demarcated by differences in the strength of purifying selection—“stronger” at high altitude (i.e., more significant instances) vs. “weaker” at low altitude (i.e., fewer significant instances). This contrasting and predictable pattern of selection suggests that the mitochondrial genome has played a key role in adaptation to high-altitude, hypoxic environments. Adaptation of nuclear and mitochondrial proteins at the level of respiratory function is thought to be stringent because of how tightly intertwined the functions of the mitochondrial and nuclear genomes are [77–80]. The compatibility between OXPHOS mitochondrial-nuclear subunits is particularly apparent in prior documented examples of population-level divergence, where it has effectively created isolating barriers leading to population structuring and speciation [81–84]. Thus, given the functional importance of the genes encoded by the mitochondrial genome, selection for optimal catalytic capacity/regulatory efficiently is expected to lead to stronger patterns of purifying selection.

Mitochondrial efficiency is rooted in the relationship of the mitochondrially-encoded subunits to their nuclear-encoded subunits, and specifically the coupling of ATP production by chemiosmosis to the electrochemical proton gradient established by the electron transport system [85]. This mito-nuclear association is thought to be coevolved with both nuclear and mitochondrial genomes evolving complementary changes in the other to ensure correct mitochondrial functioning [79, 86]. Mitochondrial efficiency has been shown to underlie adaptations to extreme environments, like at high-altitude [12, 71], as well as strongly locomotive species such as the waterfowl studied here [87–89]. However, OXPHOS is not perfectly coupled because protons can leak across the inner membrane independent of ATP synthesis [90]. Uncoupling reduces the membrane potential, leading to waste and a reduction in ATP production efficiency [91]. However, uncoupling is also important for thermogenesis, and it also has importance for reactive oxygen species (ROS) production. Since ROS production is positively correlated with the steepness of the proton gradient, uncoupling caused by proton leak can minimize ROS production and therefore prevent oxidative damage [90], which also may be a key factor influencing evolution at high altitude [25, 30].

Recent biochemical assessment of ATP production in high-altitude duck species showed respiratory capacity in high-altitude ducks was significantly associated with elevated activities of mitochondrial enzymes, and oxidative capacity [13, 28], whereupon it was suggested that high-altitude ducks may take advantage of O_2_-costly fuels (i.e., lipids), provided that sufficient tissue O_2_ supply is maintained by evolved or plastic changes throughout the O_2_ transport pathway [20, 92]. Thus, evolved changes in mitochondrial catalytic efficiency could be the reason behind such pervasive signature of purifying selection in high-altitude populations.

## Conclusions

The mitochondria is essential for producing energy in the form of ATP through the electron transport chain and its corresponding OXPHOS related complexes and enzymatic pathways, which also involve thermogenesis and ROS production. With these roles, the mitochondrial genome is frequently associated with adaptation to various environments including extreme cold environments and hypoxic environments. We assessed the role of standing genetic variation in the mitochondrial genome as it potentially facilitated adaptation to hypoxic, high-altitude environments in three Andean duck species. Based on the observed relative contributions of demography related to changes in effective population size in these species, we argue that purifying selection has played a strong role at high altitude given the consistency of results across multiple taxa with varying degrees of similarity and difference in demographic history.

Specifically, the results from the three study species suggest that there is selection on the mitogenome across species, but that the same biochemical mechanisms do not always result from the same environmental pressures, as illustrated by two of the same species, in which previous work showed evidence of both short- and long-term adaptation through the hypoxia-inducible factor pathway [22] and hemoglobin complexes [20, 75, 76]. Thus, species invade a new habitat potentially using other mechanisms but then adapt to it—i.e., speckled teal has had enough time for selection to show signs of distinct biochemical pathways, whereas more recently arrived species have not [28].

These three Andean waterfowl species successfully colonized and, are thriving in the same high-altitude environment, with purifying selection acting to maintain the OXPHOS unit’s ability to operate optimally. The signature of purifying selection on the mitogenome is especially apparent for the more deeply diverged speckled teal with the longer history of occupancy at high altitude. Specifically, a substantial increase in purifying selection resulting in decreased nucleotide diversity in this species, is likely due not only to the invasion of high-altitude niches but also due to elapsed time at altitude. These results open up additional questions associated with how purifying selection of this type influences mito-nuclear interactions, plus a general need for a greater understanding of the physiology and biochemistry associated with mitochondria function in this extreme environment. Finally, it should be noted that achieving a better understanding of the unique biochemistry and physiology of these species in this extreme environment should also be beneficial to the long-term conservation of these high Andean species, especially as factors related to mitochondrial function relate to capacity for movement and thermogenesis in a rapidly changing climate where suitable wetlands may be shifting.

## Supporting information

S1 Table Sample and assembly statistics associated with the mitochondrial genome assemblies of the three Andean duck species. (XLSX)

S2 Table Tamura-Nei genetic distance between low- and high-altitude populations of the three Andean duck species. (XLSX)

S3 Table Significant *F*_*ST*_ values in the mitochondrial genome of speckled teal. (XLSX)

S4 Table Significant instances of selection (P > 0.9) across sites using FUBAR (Fast, Unconstrained Bayesian AppRoximation) for each of the three species: Cinnamon Teal (CT), Speckled Teal (ST), Yellow Billed Pintail (YBP). Prob [P] is the posterior probability. (XLSX)

S5 Table Tajima’s D statistics calculated in MEGA for mitochondrial gene regions of three Andean duck species. (XLSX)

S6 Table Tajima’s D statistics for each OXPHOS complex within the mitochondrial genome. (XLSX)

S7 Table Tajima’s D statistics for the non-coding elements (tRNA, rRNA and dLoop) within the mitochondrial genome. (XLSX)

S1 Fig (JPG)
