# Supplementary figures and images for: High-altitude adaptation is accompanied by strong signatures of purifying selection in the mitochondrial genomes of three Andean waterfowl
Source: PLoS One. 2024 Jan 3;19(1):e0294842. doi: 10.1371/journal.pone.0294842 (PMC10763953; doi:10.1371/journal.pone.0294842)

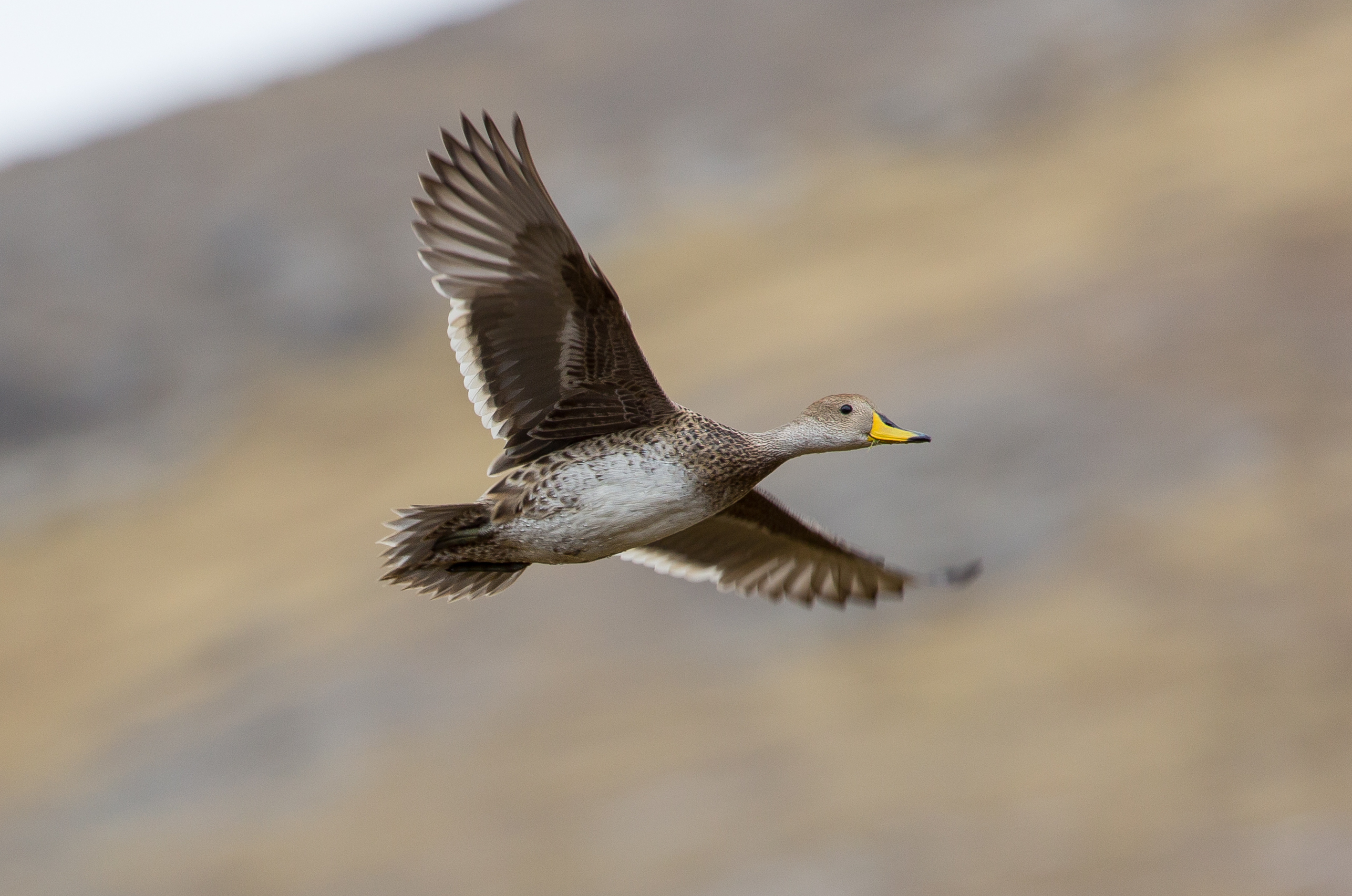

Supplement: S1 Fig — (JPG) [file pone.0294842.s008.jpg]
